# Mesonephric‐type adenocarcinomas of the ovary: prevalence, diagnostic reproducibility, outcome, and value of PAX2

**DOI:** 10.1002/2056-4538.12389

**Published:** 2024-07-06

**Authors:** Martin Köbel, Eun Young Kang, Sandra Lee, Travis Ogilvie, Tatjana Terzic, Linyuan Wang, Nicholas JP Wiebe, Zainab Al‐Shamma, Linda S Cook, Gregg S Nelson, Colin JR Stewart, Andreas von Deimling, Felix KF Kommoss, Cheng‐Han Lee

**Affiliations:** ^1^ Department of Pathology University of Calgary Calgary AB Canada; ^2^ Department of CSPH‐Epidemiology University of Colorado‐Anschutz Aurora CO USA; ^3^ Department of Oncology, Division of Gynecologic Oncology, Cumming School of Medicine University of Calgary Calgary AB Canada; ^4^ Department of Anatomical Pathology, King Edward Memorial Hospital Subiaco WA Australia; ^5^ School for Women's and Infants’ Health University of Western Australia Perth WA Australia; ^6^ Department of Neuropathology Heidelberg University Hospital and CCU Neuropathology DKFZ Heidelberg Germany; ^7^ Department of Pathology Heidelberg University Hospital Heidelberg Germany; ^8^ Department of Laboratory Medicine and Pathology University of Alberta Edmonton AB Canada

**Keywords:** mesonephric, mesonephric‐like, PAX2, GATA3, TTF1

## Abstract

Mesonephric‐type (or ‐like) adenocarcinomas (MAs) of the ovary are an uncommon and aggressive histotype. They appear to arise through transdifferentiation from Müllerian lesions creating diagnostic challenges. Thus, we aimed to develop a histologic and immunohistochemical (IHC) approach to optimize the identification of MA over its histologic mimics, such as ovarian endometrioid carcinoma (EC). First, we screened 1,537 ovarian epithelial neoplasms with a four‐marker IHC panel of GATA3, TTF1, ER, and PR followed by a morphological review of EC to identify MA in retrospective cohorts. Interobserver reproducibility for the distinction of MA versus EC was assessed in 66 cases initially without and subsequently with IHC information (four‐marker panel). Expression of PAX2, CD10, and calretinin was evaluated separately, and survival analyses were performed. We identified 23 MAs from which 22 were among 385 cases initially reported as EC (5.7%) and 1 as clear cell carcinoma. The interobserver reproducibility increased from fair to substantial (*κ* = 0.376–0.727) with the integration of the four‐marker IHC panel. PAX2 was the single most sensitive and specific marker to distinguish MA from EC and could be used as a first‐line marker together with ER/PR and GATA3/TTF1. Patients with MA had significantly increased risk of earlier death from disease (hazard ratio = 3.08; 95% CI, 1.62–5.85; *p* < 0.0001) compared with patients with EC, when adjusted for age, stage, and p53 status. A diagnosis of MA has prognostic implications for stage I disease, and due to the subtlety of morphological features in some tumors, a low threshold for ancillary testing is recommended.

## Introduction

There has been a surge in studies of mesonephric‐like adenocarcinomas involving the endometrium and ovary in recent years [1]. Due to a lack of an association with mesonephric remnants, McFarland *et al* proposed the term mesonephric‐like adenocarcinoma to separate them from mesonephric‐type adenocarcinoma (MA) of the uterine cervix [2]. In 2020, this entity received its own ICD‐O code (9111/3) [3]. Evidence including associated or admixed Müllerian lesions (e.g. atypical endometrial hyperplasia, endometriosis, endometrioid neoplasm, and low‐grade serous carcinoma) and shared clonal relationship support a Müllerian origin of these tumors with Wolffian/mesonephric transdifferentiation, rather than a true mesonephric origin [4, 5, 6, 7, 8]. The phenomenon of transdifferentiation mostly from endometrioid lesions would also explain the morphological overlap with endometrioid carcinomas (ECs) resulting in diagnostic problems. McFarland *et al* initially described this entity as an ‘unusual variant of endometrioid carcinoma’ [2], leading to a dispute whether this is a truly independent entity [9]. Mesonephric‐like adenocarcinomas of the ovary and the uterine corpus and MA of the uterine cervix share identical phenotypes, *KRAS* driver mutations, and overlapping proteomic and DNA methylation profiles, the latter being distinct from other Müllerian histotypes and potentially somatically acquired during transdifferentiation [10, 11, 12]. Therefore, we proposed using the same term of ‘mesonephric‐type’ adenocarcinoma (MA) and the ICD‐O code 9110/3 for tumors from cervical, endometrial, and ovarian sites, regardless of the cell of origin [12, 13].

A four‐marker immunohistochemical (IHC) panel of GATA3, TTF1, ER, and PR was used in the discovery of MA and represents a valuable ancillary test [1, 2]. PAX2 is another marker of benign mesonephric cell lineage but its diagnostic utility in the context of MA has not been evaluated [14]. The objectives of our study were first to assess the prevalence of MA in large retrospective cohorts of ovarian carcinomas using a combination of IHC screening and morphological review. Second, we evaluated the interobserver reproducibility based on morphology alone and morphology plus IHC to refine the diagnostic approach. Third, we compared survival and biomarker expression of MA and EC.

## Materials and methods

### Study cohorts

An overview of the study flow is provided in supplementary material, Figure S1. We gathered 1,537 ovarian epithelial neoplasms from two existing population‐based ovarian carcinoma cohorts [Ovarian Cancer in Alberta and British Columbia (OVAL‐BC), *n* = 804 and Alberta Ovarian Tumour Type (AOVT), *n* = 536] and from previously described consecutive hospital‐based series (*n* = 197) [15, 16, 17]. The OVAL‐BC study recruited incident cases from cancer registries of two Canadian provinces diagnosed between 2001–2012 (BC) and 2005–2011 (AB) [18]. The AOVT study identified ovarian carcinomas from the Alberta Cancer Registry diagnosed between 1978 and 2010 [19]. Duplicate patients in the OVAL‐BC and AOVT cohorts were excluded. All cases were subjected to an IHC‐integrated review to confirm or reclassify histotypes [18, 19]. Previously assessed IHC markers (ER/PR, PMS2/MSH6, p53, WT1, PAX8, and ARID1A) were used for correlative analyses [18, 19, 20, 21, 22, 23]. PMS2/MSH6 and p53 were used to assign the mismatch repair deficient (MMRd) and p53 abnormal (p53abn) molecular subtypes for EC [24]. *POLE* mutation status was not available; hence, MMR proficient and p53 normal EC were considered of no specific molecular profile (NSMP). ECs were graded using FIGO grade [25]. All cohorts received local ethics approval (HREBA.CC‐16‐0161, HREBA.CC‐16.0159, HREBA.CC‐16.0371, HREBA.CC‐21‐0362, and HREBA.CC‐19‐0444).

### Immunohistochemistry and interpretation

Sections of 4‐μm thickness obtained from tissue microarrays (TMAs) were used for IHC on a Dako Omnis autostainer (Agilent Technologies, Santa Clara, CA, USA) with onboard heat‐induced epitope retrieval, followed by antibody incubation and use of Dako EnVision FLEX (Agilent Technologies). The antibody clones, suppliers, dilutions, and Dako specific protocols were as follows: GATA3 (L50‐823, Biocare, 1/400, H20‐10M‐20), TTF1 (SPT24, Leica, 1/200, H20‐X‐20), PAX2 (EP235, Bio SB, 1/50, H30‐10R‐30), CD10 [56C6, DAKO, ready‐to‐use (RTU), H30‐X‐30], and Calretinin (Dak‐Calret1, Dako, RTU, H25‐X‐25). Markers were scored in a three‐tier system based on staining distribution and categorized as 0 = absent, 1 = focal (staining in 1–50% of tumor cells), and 2 = diffuse (staining >50%). The results were dichotomized as either absent or present, except for PAX2, where diffuse was considered normal retained versus abnormal being reduced or absent. ER and PR were paired and the higher expression value of the two markers was used to represent the ER/PR combination. From the four‐marker panel of GATA3, TTF1, and ER/PR, four IHC groups were created: (1) MA‐IHC profile, defined as GATA3 and/or TTF1 expression with complete absence of ER/PR; (2) EC‐IHC profile, defined as absence of GATA3 and TTF1 with at least focal expression of ER/PR; (3) IHC double‐negative, which were negative for Wolffian/mesonephric markers (GATA3, TTF1) and Müllerian markers (ER/PR); and (4) IHC double‐positive, which were positive GATA3 and/or TTF1 with at least focal ER/PR.

### Identification of ovarian MA in retrospective cohorts

To identify MA, we used a two‐step process. The first step was the application of a four‐marker IHC screen (GATA3, TTF1, ER, and PR) followed by a morphologic review of tumors with an MA‐IHC profile. Since almost all MA cases detected by the IHC screen were previously diagnosed as EC, as a second step, we conducted a morphologic review of the remaining 369 EC, followed by integration of IHC profile (supplementary material, Figure S1). The latter was performed on two representative full sections that were selected during previous full slide reviews [18, 19] independently by two pathologists (MK and ZA) with the knowledge of the IHC profile. Both did not participate in the interobserver reproducibility study and achieved consensus in discordant instances at a multiheaded microscope.

### Interobserver reproducibility

A reproducibility set of 66 cases consisting of EC and MA, enriched for the latter, was compiled and one representative digital slide per case was scanned at ×40 magnification using an Aperio scanner (Leica Biosystems, Vista, CA, USA). Fourteen MAs were previously assessed for global DNA methylation profiles using the Illumina Infinium EPIC (850K) BeadChip (Illumina, San Diego, CA, USA) [12]. Five gynecologic subspecialty pathologists reviewed the digital slides, blinded to clinical information, and categorized them into EC or MA in an initial round of morphology‐based assessment. The following description for MA was provided [1]: ‘the characteristic low power architecture of MA is a compact, blue‐appearing proliferation of small or long tubules with intraluminal eosinophilic (PAS+, Alcian blue negative) secretions’; however, the latter can be a focal finding. In addition, a variety of architectural patterns is also characteristic for MA including glandular, solid/spindled, papillary, trabecular, retiform, sieve‐like, and chorded‐hyalinized. The cell shape is cuboidal rather than columnar as seen in EC. The nuclear atypia is generally low to moderate with papillary thyroid carcinoma‐like nuclear features such as open/vesicular chromatin, nuclear overlapping, grooves, angulated nuclear contours/indentations, and inconspicuous nucleoli. Mitotic activity is usually conspicuous. Squamous or mucinous differentiation may be seen in EC but should be absent in MA except for circumstances of admixed MA and EC. Subspecialty pathologists were also asked whether they would order IHC and if they indicated yes, results for the four‐marker IHC panel of GATA3, TTF1, ER, and PR (but not the actual IHC slides) were provided. They then had the opportunity to revise their diagnosis based on the IHC results.

### Morphological feature review of MA versus EC

A subset of 22 MAs and 71 ECs (52 EC NSMP, 6 EC MMRd, and 13 EC p53abn) underwent detailed morphological review from two representative whole H&E sections evaluating the following features: low power color (blue versus pale), presence of a background adenofibroma, psammoma bodies, number of architectural patterns, types of architectural patterns (sieve‐like/cribriform/microcystic, glomeruloid, glandular/pseudoendometrioid, ductal/slit‐like angulated, long tubular, trabecular, solid/spindled/nested, and papillary/villoglandular), the presence of colloid‐like luminal material, cytologic/nuclear features (nuclear crowding/overlapping, prominent nucleoli, dense, vesicular, or open chromatin, columnar cell shape with abundant cytoplasm, cuboidal cell shape with scant cytoplasm), and mitotic count per 10 high‐power fields. Squamous differentiation, mucinous differentiation, cytoplasmic clearing, and cilia were described as conspicuous, focal, or absent.

### Statistics

Categorical data were compared using Pearson's chi‐squared test and continuous data using a Student's *t*‐test or analysis of variance of means. Bonferroni correction was used to correct for multiple testing. For paired interobserver reproducibility, Cohen's kappa coefficient as well as percentage agreement was calculated and reported as an average and range over the five subspecialty pathologist pairs. Nominal logistic regression modeling was used to calculate an area under the curve with receiver operator characteristics for morphological features in combination. Recursive partitioning was performed to establish hierarchy for IHC markers. Kaplan–Meier survival analyses were performed to estimate 5‐year survivals with disease‐specific survival as the endpoint. The follow‐up period was right censored at 10 years. Differences were assessed by a log‐rank test. Cox proportional hazards regression models were applied to estimate hazard ratios (HRs) with 95% confidence intervals (CIs). Multivariate Cox regression models were adjusted for age (continuous), stage (I versus II–IV), and p53 status (normal versus abnormal). JMP17.0.0.0 was used for statistical analyses.

## Results

### Identification of ovarian MA in retrospective cohorts

**Figure 1:**
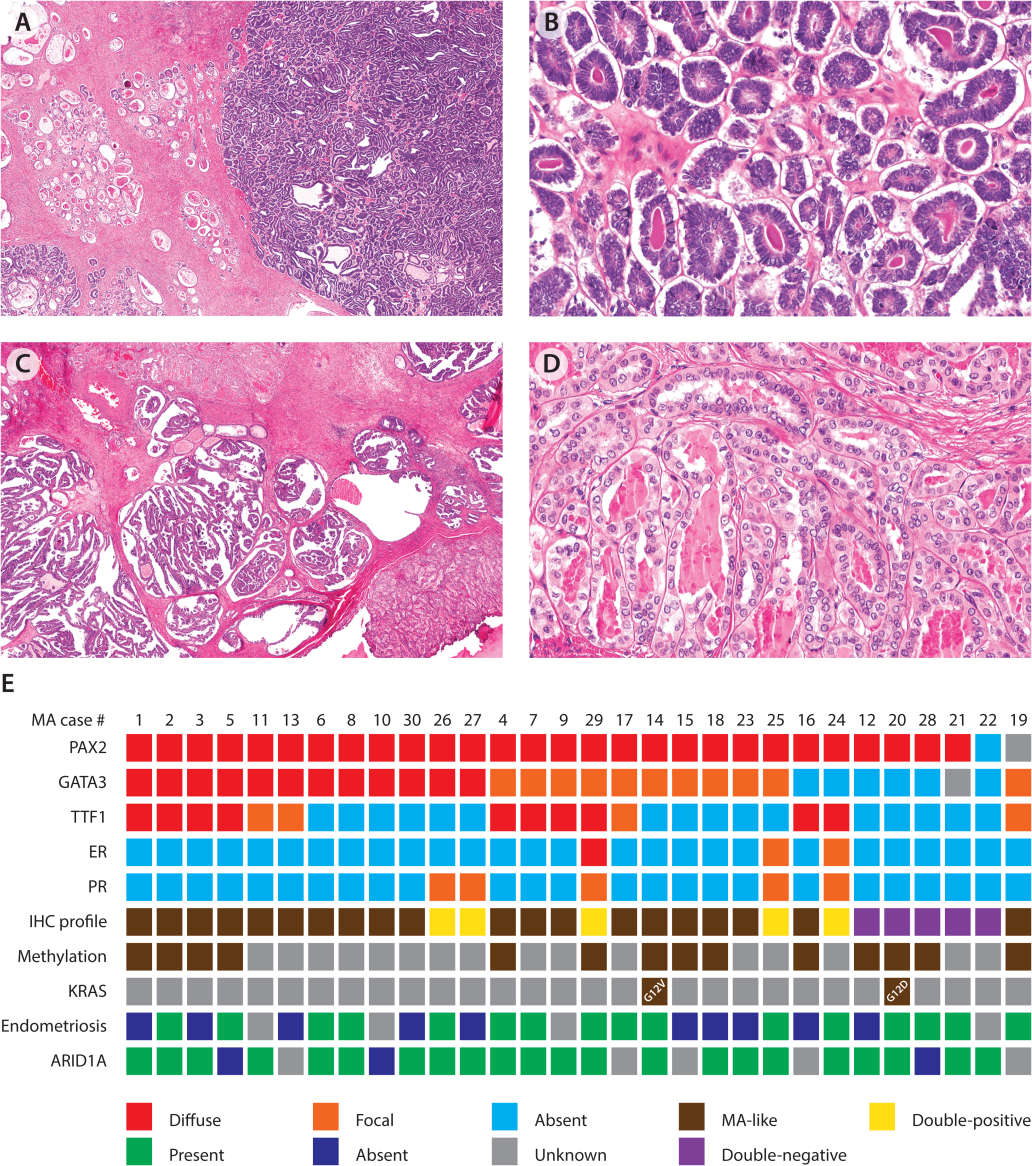
Examples of classic mesonephric‐type adenocarcinoma (MA) with a blue low‐power appearance in a pink collagenous background in (A) and (C) with an admixture of architectural patterns including (A) dense tubules and sieve‐like and (B) papillary and tubular. In corresponding higher power images (C and D), eosinophilic luminal material is conspicuous. There is nuclear overlapping in (C) and open chromatin, grooves, and angulated contours/indentations are apparent in (D). (E) Heat map showing biomarker characteristics of the MA cases as well as presence of associated endometriosis. The detailed ER/PR expression reported as % distribution and (three‐tier intensity) for the five positive MAs were as follows: MA#29 ER 100%(3)/PR 1%(1); MA#24 ER 40%(1)/PR 1%(2); MA#25 ER 5%(1)/PR 20%(3); MA#26 ER 0%/PR 10%(2); MA#27 ER 0%/PR 5%(2).

First, a four‐marker IHC screen was performed on 1,537 ovarian epithelial neoplasms, and the expression of GATA3, TTF1, ER, and PR across histotypes is shown in supplementary material, Table S1. EC showed the highest frequency of GATA3 expression (37/384, 9.6%; 5.7% focal, 3.9% diffuse) and TTF1 (35/384, 9.1%; 2.9% focal, 6.2% diffuse). The MA‐IHC profile was present in 45/1,537 (2.9%) across all histotypes. Of the 45 tumors with an MA‐IHC profile, 13 (28.8%) were deemed to be MA on morphologic review (12 initially classified as EC, 1 initially classified as clear cell carcinoma). The remaining 32 MA‐IHC profiles were confirmed by morphological review as 4 ECs, 22 clear cell carcinomas, 5 mucinous carcinomas, and 1 mucinous borderline tumor. Morphological review of the remaining 369 ECs with the knowledge of their IHC profile identified an additional 20 instances with morphological MA features. By integration of their IHC profile, eight EC‐IHC were classified as EC and five IHC double‐negative cases as MA. Of the remaining seven IHC double‐positive cases, three had high ER/PR expression and four had low ER/PR expression. Although we used GATA/TTF1/ER/PR for our primary IHC screen, we separately assessed PAX2 as a potential mesonephric marker. PAX2 was expressed in normal Müllerian tissue including endometrium, fallopian tube, and mesonephric remnants (supplementary material, Figure S2). PAX2 showed normal expression in almost all MA versus 8.0% of high‐grade serous carcinomas and 14.6% of initial EC (Figure 1 and supplementary material, Table S2). Therefore, based on PAX2, five IHC double‐positive cases were ultimately classified as MA (four low ER/PR and PAX2 normal, one high ER/PR and PAX2 normal, supplementary material, Figure S3) and two as EC (both high ER/PR and PAX2 abnormal). The prevalence of MA among those initially classified as EC was 5.7% (22/385, supplementary material, Figure S1). We identified another 7 recently diagnosed MA during a period where 62 ECs diagnoses were made resulting in a total of 30 MAs from which 14 had previously been subject to DNA methylation profiling [12]; all 14 showed epigenetic profiles in keeping with mesonephric differentiation (Figure 1).

### Interobserver reproducibility

One representative digital slide per case from the review set of 66 tumors was evaluated by five subspecialty pathologists (supplementary material, Table S2). The diagnostic agreement between MA and EC reached a fair kappa coefficient of 0.376 (range: 0.209–0.602, supplementary material, Table S3). A diagnosis of MA was favored on average in 10/66 (15.2%, range: 5–16) and requested the four‐marker IHC panel on average in 38/66 (57.6%, range: 25–46) cases (supplementary material, Table S4). After integration of IHC (GATA3/TTF1/ER/PR) results, the interobserver agreement improved to a substantial kappa of 0.727 (range: 0.655–0.852, *p* = 0.00017, supplementary material, Table S3). Changes were made on average in 8 cases (range: 4–13), in the majority toward MA (average 6, range: 3–11) and less toward EC (average 2, range: 1–5) resulting in an average MA diagnosis in 21.2% (14/66 cases, range: 11–19, supplementary material, Table S4). By integrating IHC, consensus among the five subspecialty pathologists increased from 43/66 (65.2%, 43 ECs, 0 MA) to 53/66 (80.3%, 46 ECs, 7 MAs).

### Persistent diagnostic issues

**Figure 2:**
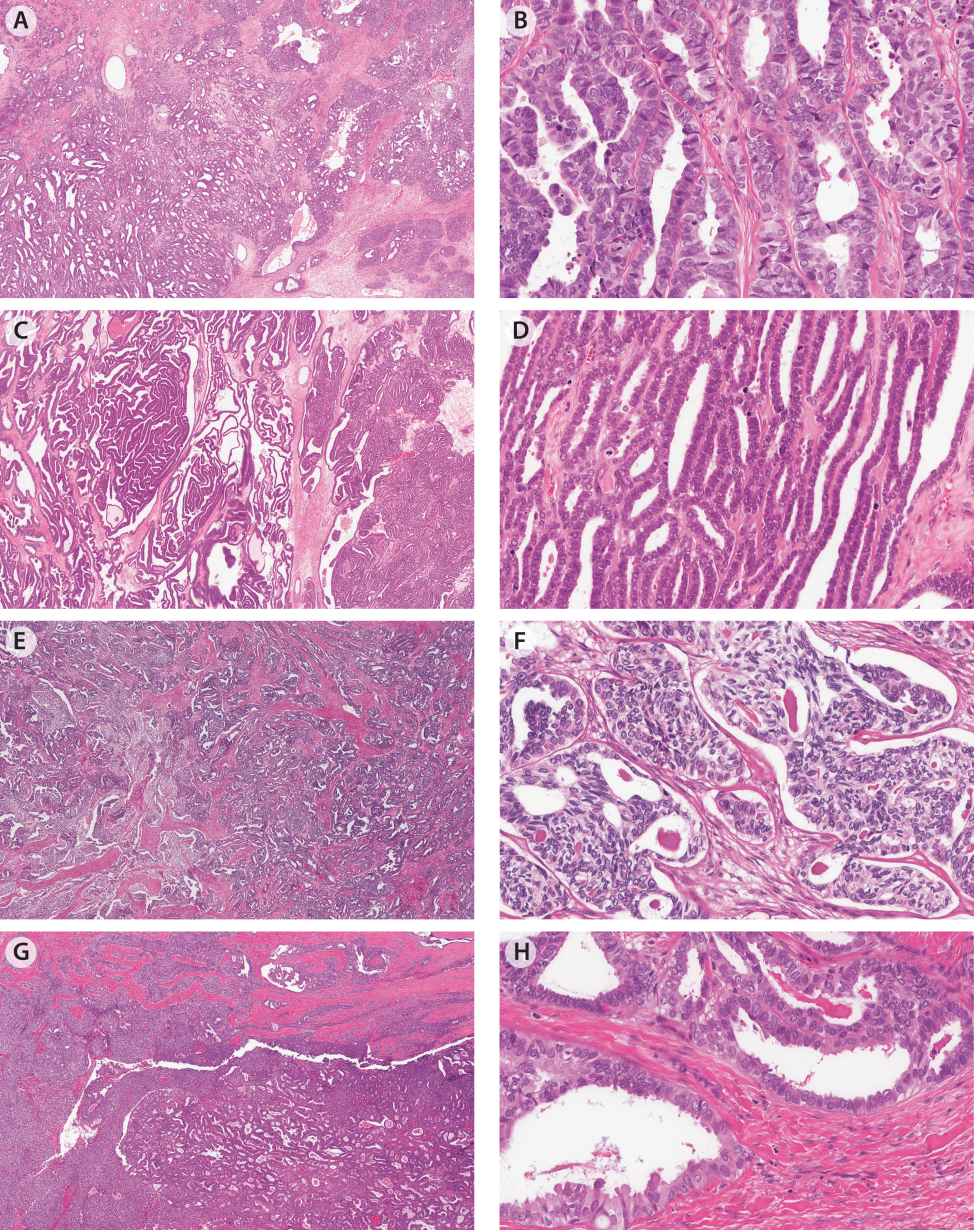
Cases from the reproducibility set with persistent issues where immunohistochemistry was not ordered. (A and B) MA#02 with angulated ducts and cribriform architecture. Eosinophilic luminal material is absent, and the cell shape is more cuboidal. (C and D) MA#03 with more typical long tubular and papillary architectures and cuboidal cell shape. (E and F) MA#04 with nested and spindled architecture and focal eosinophilic luminal material. (G and H) MA#05 with nested/solid architecture and angulated ducts with focal eosinophilic luminal material. Some columnar cells with apical secretions and focal cilia are noted but the predominant cell shape is cuboidal.

**Figure 3:**
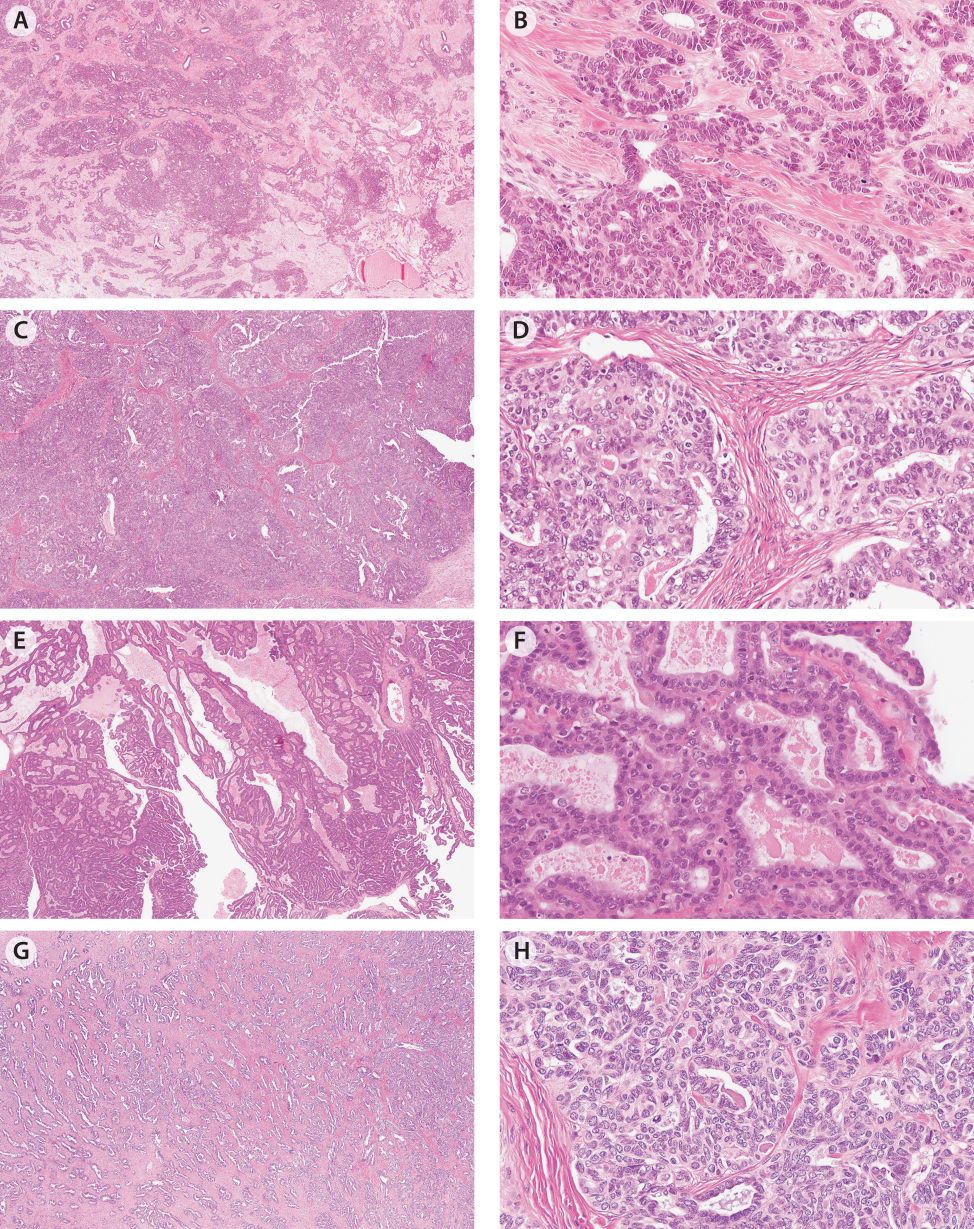
Cases from the reproducibility set with persistent issues of immunohistochemistry (IHC) integration including cases that did not show a mesonephric‐type IHC profile. (A and B) MA#12 with nested/solid architecture and angulated ducts as well as focal eosinophilic luminal material; double‐negative IHC profile but MA methylation profile and normal retained PAX2 expression. (C and D) MA#28 with nested/solid architecture and angulated ducts with focal eosinophilic luminal material; double‐negative IHC profile but MA methylation profile and normal retained PAX2 expression. (E and F) MA#29 with papillary and angulated ductal architecture and cuboidal cell shape and conspicuous eosinophilic luminal material, double‐positive IHC profile (diffuse ER expression) and MA methylation profile as well as normal retained PAX2 expression. (G and H) MA#18 with pale low‐power appearance, angulated ductal architecture, cuboidal cell shape, eosinophilic luminal material and typical nuclear features. The case had a MA‐IHC profile with only focal GATA3 expression.

Even after IHC integration using the four‐marker panel, diagnostic discordances remained in 13 cases with 1/5 subspecialty pathologists deviating from the majority in 9 and 2/5 subspecialty pathologists in 4. Of these 13, 11 had previously been subject to DNA methylation profiling [12], all of which showed epigenetic profiles in keeping with mesonephric differentiation. Of note, this included four cases with a majority diagnosis of EC (supplementary material, Table S2). Diagnostic discordance occurred when IHC data were not requested (nine instances affecting nine cases, Figure 2), suggesting some MAs have subtle morphological features and/or morphological features were not well appreciated. Further, the integration of the IHC results with morphology caused difficulties in 14 instances affecting 7 cases. The latter consisted of IHC double‐negative (*n* = 4), IHC double‐positive (*n* = 2), and one case with an MA‐IHC profile (Figure 3). One double‐positive tumor with diffuse ER expression was considered MA by a minority of subspecialty pathologists, a diagnosis supported by DNA methylation profiling results (supplementary material, Figure S3).

### Morphological feature review of MA versus EC

Among the morphological features assessed on 22 MAs and 71 ECs, 8 were not significantly different between the two histotypes, and these included solid/spindled/nested and papillary/villoglandular architecture as well as cytoplasmic clearing, cilia, chromatin pattern, mitotic count and psammoma bodies (supplementary material, Table S5). After a Bonferroni correction for multiple testing, eight features remained significantly different between MA and EC, and these included blue low‐power color, colloid‐like luminal material, three or more architectural patterns, sieve‐like/cribriform/microcystic and glomeruloid architectures, columnar cell shape with abundant cytoplasm or cuboidal cell shape with scant cytoplasm, and nuclear crowding. As columnar and cuboidal cell shape were nearly mutually exclusive, only cuboidal cell shape was included in a nominal logistic regression model together with the other significant features yielding an area under the curve of 0.998 in distinguishing MA from EC with cuboidal cell shape with scant cytoplasm and blue low‐power color being the strongest contributors to the model.

### Clinicopathological features of MA

**Figure 4:**
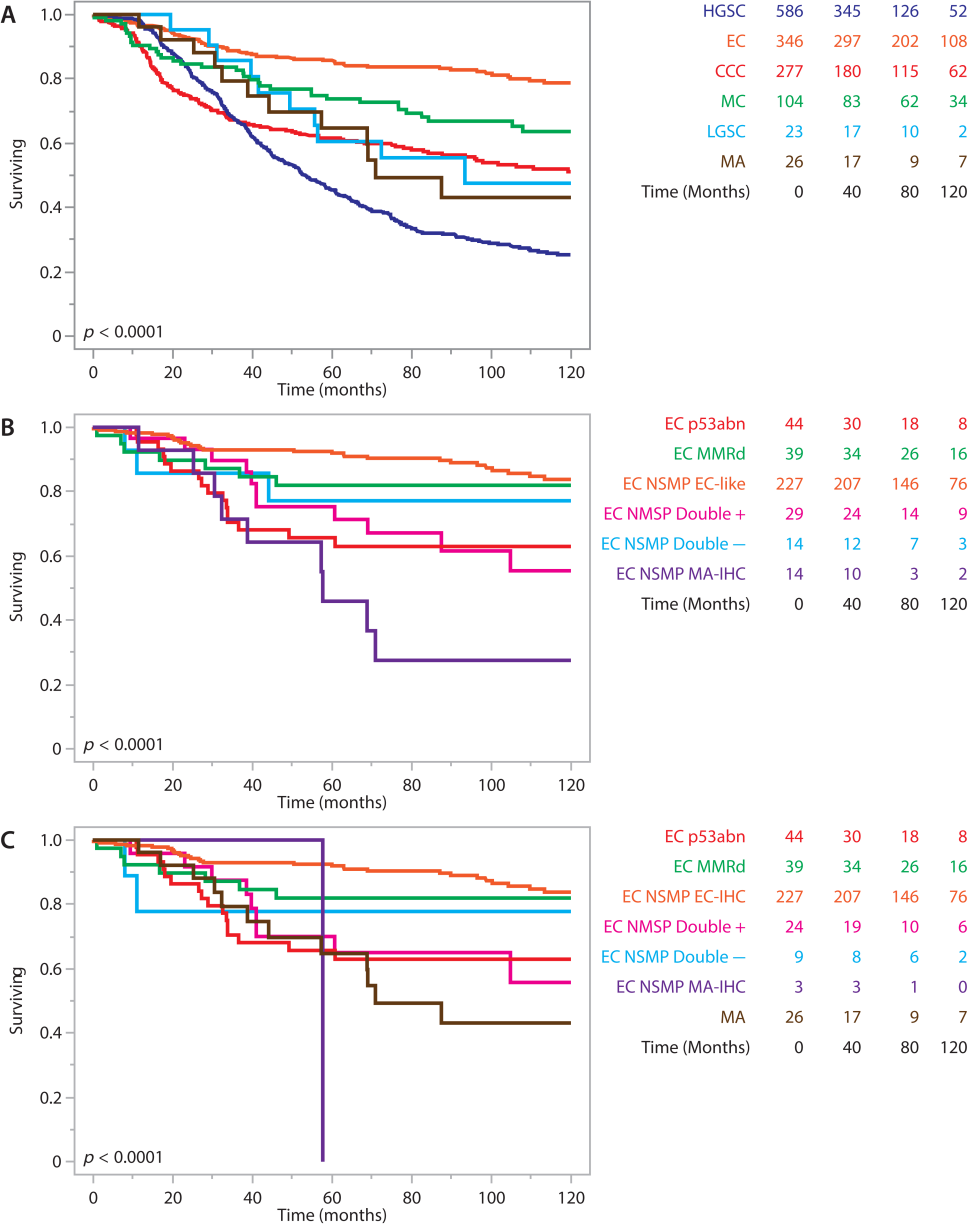
Kaplan–Meier survival analysis; *p* value – log‐rank test. (A) Comparison of mesonephric‐type adenocarcinomas (MA) to other histotypes [low and high grade serous, endometrioid (EC), clear cell, and mucinous carcinomas]. (B) Grouped immunohistochemical (IHC) profiles of EC (initial diagnosis). (C) Comparison of MA to grouped IHC profiles of EC (diagnosis after MA reclassification). Tables on the right indicate numbers at risk.

The clinicopathological features of 30 MAs and 363 ECs were compared (Table 1). Patients with EC were on average 8 years younger than those with MA. Associated endometriosis occurred at a similar rate. Despite a similar stage distribution, the 5‐year survival for patients with MA was lower (64.8%) compared with EC (85.7%) but slightly better than that of high‐grade serous carcinoma patients (45.4%, Figure 4A). In multivariable analysis adjusted for age, stage, and p53 status, patients with MA had an increased risk of earlier death (HR = 3.08, 95% CI: 1.62–5.85, *p* < 0.0001) compared with the reference EC group. This was a slightly higher risk than p53 abnormal EC (HR = 2.32, 95% CI: 1.30–4.14, supplementary material, Table S6). The survival difference between MA and EC NSMP remained significant in a stratified analysis for stage I disease (log‐rank *p* = 0.0002, supplementary material, Figure S4).

**Table 1:** Clinicopathological characteristics of mesonephric‐type and endometrioid carcinomas included in the study (*N* = 393)

	MA (*N*, %)		EC (*N*, %)		*p* value
*N*	30		363		
Age					
Mean, SE (years)	61.9	2.2	54.1	0.6	0.0008
Stage					
I (limited to ovary)	17	56.7	190	56.7	0.996
II–IV (beyond ovary)	13	43.3	145	43.3	
Unknown	0		28		
Tumor size					
Mean, SE (cm)	12.6 (1.2)		11.9 (0.4)		0.63
Survival					
5‐year survival, SE	64.8 (3.5)		85.7 (1.4)		0.0002
Grade					
1	11	36.7	147	69.0	0.001
2	13	43.3	32	15.0	
3	6	20.0	34	16.0	
Unknown	0		150		
Endometriosis					
Present	16	61.5	127	58.0	0.73
Absent	10	38.5	92	42.0	
Unknown	0		144		

EC, endometrioid carcinoma; MA, mesonephric‐type adenocarcinoma; SE, standard error.

### Biomarker expression of MA compared to EC

**Figure 5:**
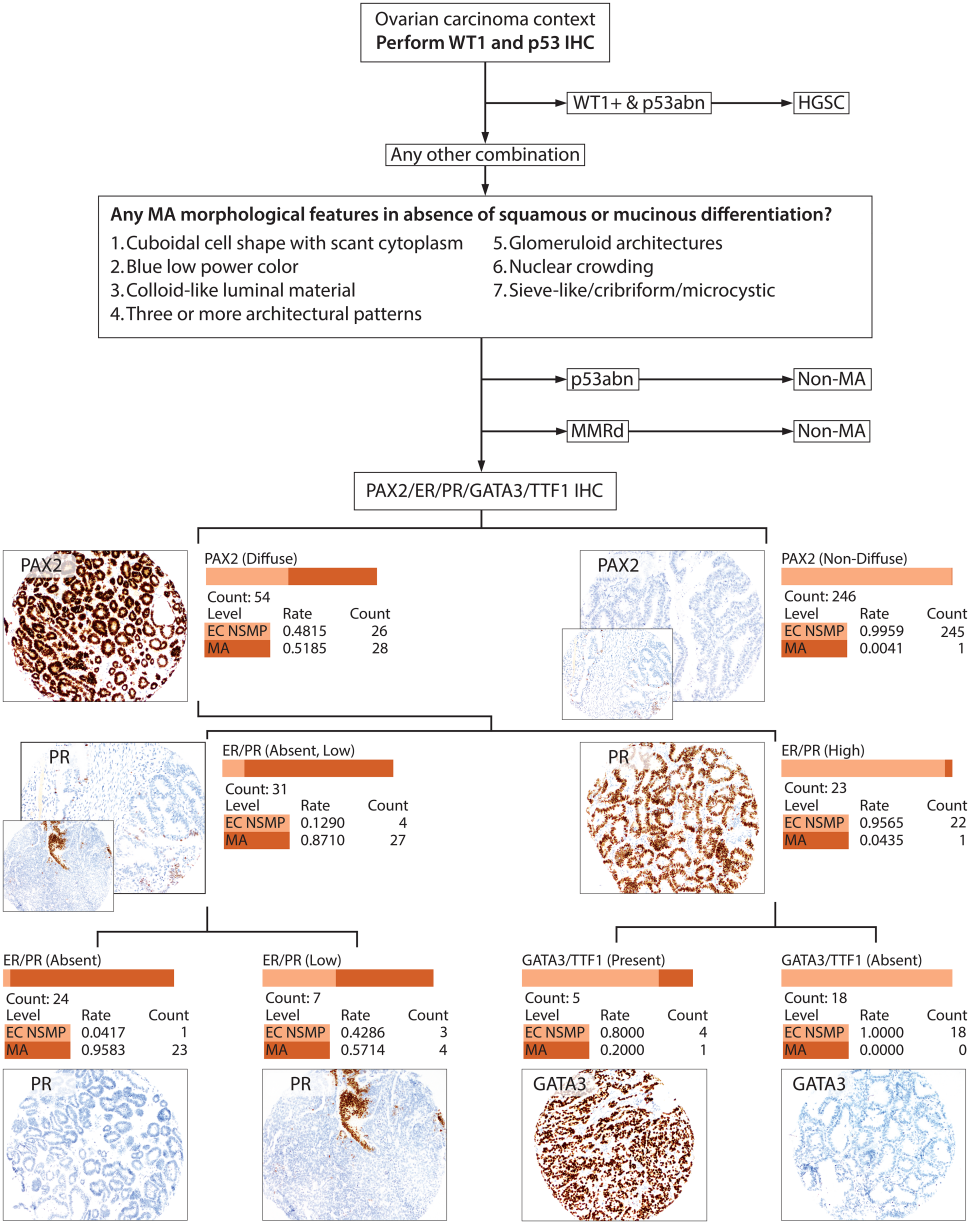
Hierarchical decision tree using combined morphologic and immunohistochemistry (IHC)‐based identification of mesonephric‐type adenocarcinoma (MA). First, a combination of WT1+ and p53 abnormal is highly specific for high‐grade serous carcinoma (HGSC). Second, there are typical morphological features of MA; however, they can overlap with endometrioid carcinomas (EC). Third, to distinguish EC from MA, p53abn and MMRd molecular subtypes of EC are generally inconsistent with MA. Fourth, the most efficient approach to distinguish MA from EC, NSMP molecular subtype (alternative approach without PAX2 is shown in supplementary material, Figure S5).

Table 2 compares the expression of selected IHC markers in MA with EC, with significant differences in GATA3, TTF1, PAX2, ER, PR, MMR proteins, p53, and WT1. Notably, the expression of CD10 and calretinin was not significantly different. PAX2 showed both high sensitivity (96.6%) and high (90.4%) specificity for MA when using a cutoff of >50% of tumor cells for normal expression. Using hierarchical partitioning with normal versus abnormal PAX2 expression, combined ER/PR status, and either GATA3 or TTF1 positivity achieved >98% precision (*R* ^2^, 0.822) in distinguishing MA from EC. Figure 5 shows a proposed morphology and IHC‐based diagnostic decision algorithm (alternative approach without PAX2 shown in supplementary material, Figure S5).

**Table 2:** Expression of select immunohistochemical markers in mesonephric‐type and endometrioid carcinomas (*N* = 393)

	MA (*N*, %)		EC (*N*, %)		*p* value
*N*	30		363		
GATA3					
Present	23	79.3	21	5.8	<0.0001
Absent	6	20.7	342	94.2	
Unknown	1		0		
TTF1					
Present	14	46.7	25	6.9	<0.0001
Absent	16	53.3	337	93.1	
Unknown	0		1		
GATA3/TTF1					
Present	25	86.2	40	11.0	<0.0001
Absent	4	13.8	323	89.0	
Unknown	1		0		
ER/PR					
High	1	3.3	320	88.2	<0.0001
Low	4	13.3	20	5.5	
Absent	25	83.4	23	6.3	
Unknown	0		0		
4‐marker IHC profile					
EC	0	0.0	287	79.1	<0.0001
Double positive	5	16.7	36	9.9	
ER/PR low	0	0.0	17	4.7	
ER/PR negative	5	16.7	19	5.2	
MA	20	66.6	4	1.1	
MMRd					
Present	0	0.0	43	12.5	0.043
Absent	29	100.0	302	87.5	
Unknown	1	100.0	18		
p53					
Abnormal	0	0.0	46	12.9	0.039
Normal	29	100.0	311	87.1	
Unknown	1		6		
WT1					
Present	0	0.0	41	11.5	0.016
Absent	29	100.0	315	88.5	
Unknown	1		7		
PAX8					
Present	17	94.4	130	76.0	0.074
Absent	1	5.6	41	24.0	
Unknown	12		192		
ARID1A					
Present	19	86.4	245	71.8	0.14
Absent	3	13.6	96	28.2	
Unknown	8		22		
CD10					
Present	17	77.3	122	58.4	0.085
Absent	5	22.7	87	41.6	
Unknown	8		154		
Calretinin					
Present	3	15.0	10	4.8	0.062
Absent	17	85.0	197	95.2	
Unknown	10		156		
PAX2					
Normal retained	28	96.6	34	9.6	<0.0001
Abnormal	1	3.4	321	90.4	
Unknown	1		8		
CTNNB1					
Membranous	10	83.3	177	58.0	0.081
Nuclear	2	16.7	128	42.0	
Unknown	18		58		
PTEN					
Present	16	100	87	71.3	0.013
Absent	0	0	35	28.7	
Unknown	14		241		

EC, endometrioid carcinomas; MA, mesonephric‐type adenocarcinoma; MMRd, mismatch repair deficiency.

### Survival analysis within EC

Within EC, we performed survival analysis comparing the different IHC profiles also including the molecular subtypes (p53abn, MMRd, and NSMP). In the original EC cohort, an MA‐IHC profile as well as p53 abnormal molecular subtype was associated with a shorter survival, whereas a double‐positive and double‐negative IHC profile showed an intermediate prognosis compared with the favorable EC‐IHC profile and MMRd molecular subtype (Figure 4B). After removing MA from the EC pool, EC NSMP with a double‐positive IHC profile, EC p53 abnormal and MA all had a similar survival (Figure 4C). We also noted that for individual markers, significant unfavorable prognostic associations for GATA3, TTF1, and combined ER/PR status remained for the revised EC NSMP cohort even after MA was removed (supplementary material, Figure S6). However, this did not apply for normal PAX2, which was significantly associated with unfavorable prognosis in the original EC cohort, but not after MA was removed. In contrast, the nine EC NSMP with morphological features of MA but an EC‐IHC profile had a favorable outcome with no patient dying of disease (supplementary material, Figure S7).

### Survival analysis within MA

Finally, we performed exploratory survival analysis within the 26 MAs with available outcome data. Surprisingly, stage was not associated with survival, and patients with MA confined to the ovary had a 5‐year survival of 65.1% compared with 61.9% seen in MA with spread beyond the ovary (log‐rank *p* = 0.24, supplementary material, Figure S8). While grade and GATA3 status were not prognostic, there was a significant association with shorter survival for TTF1 expressing MA (log‐rank *p* = 0.0077, supplementary material, Figure S8).

## Discussion

From a diagnostic perspective, our study shows that MAs are underrecognized when classic morphological features are not identified. There were also difficulties in integrating morphological features with certain IHC profiles. Furthermore, IHC profiles alone are of prognostic significance. We recommend a low threshold for morphological features of MA to order an ancillary IHC marker panel consisting of PAX2, ER/PR, and GATA3/TTF1.

The current understanding is that MA develops through transdifferentiation from predominantly endometrioid Müllerian lesions, which is further supported by a high frequency of associated endometriosis in MA (62% in the current study, which is nearly identical to the rate of 63% reported in a recent study on extrauterine MA [26]) as well as the loss of ARID1A expression observed in a subset of MA in the current study. The process of transdifferentiation from a Müllerian to a mesonephric phenotype, however, may not be a sudden categorical switch but rather a continuum, which would explain the diagnostic challenges between MA and EC. This is exemplified in our study by cases with EC phenotype by light microscopy but GATA3/TTF1 expression and varying levels of ER/PR expression (double‐positive for mesonephric and Müllerian markers) showing a similar prognosis to MA. Double‐positive EC occurred at a similar frequency to cases reclassified as MA. GATA3 and TTF1 remained prognostic as individual markers in EC even after MAs were removed. This suggests that these markers are an early indication of mesonephric transdifferentiation in tumors that otherwise largely retain Müllerian features. Further in‐depth study of these double‐positive (i.e. positive for mesonephric and Müllerian markers) cases focusing on *KRAS* mutation status, DNA methylation, and copy number profiles may shed light on the question of how much ER/PR positivity is acceptable in MA. Notably, we observed one double‐positive case with >50% ER/PR expression in the reproducibility set, which by DNA methylation profiling clustered with MA [12]. Considering the methylation profile as a well‐conserved marker of cell lineage, this most likely represents a true MA. Euscher *et al* also accepted 3/33 cases with >50% ER expression as ovarian MA [26]. Comparing the methylation profile of these cases with bona fide MA and EC might reveal examples of an intermediate mesonephric‐like state of transdifferentiation between Müllerian and mesonephric differentiation.

On the other hand, samples that showed morphological features of MA but an EC‐IHC profile and favorable prognosis indicate that ancillary IHC is important to correctly diagnose these cases. In general, however, MA morphology was underrecognized in the reproducibility set and the significant increase in interobserver reproducibility when integrating IHC information supports the notion that ancillary IHC is required for an MA diagnosis. According to our hierarchical decision tree, cases with normal retained PAX2 expression and absence of ER/PR with an appropriate morphological phenotype are almost certainly MA. Despite the long‐established knowledge that PAX2 is a marker of mesonephric cell lineage with expression in benign mesonephric remnants [14], and case reports of PAX2 expression in MA [27, 28], it is somewhat surprising that PAX2 has not been further studied as a potential diagnostic marker for MA, particularly since it has been shown that PAX2 is lost in endometrioid neoplasms including the precursor stage of atypical endometrial hyperplasia [29]. Herein, we show that PAX2 is the most sensitive and specific single marker to distinguish MA from EC based on a large number of cases. In fact, almost all MAs showed strong diffuse, normal PAX2 expression in TMA cores. GATA3 and TTF1 demonstrated limited sensitivity in our study with 79.3% and 46.7%, respectively, which for GATA3 is in line with prior studies (90.5%, 38/42) [2, 26, 30]. However, the frequency of TTF1 expression in our study was lower compared with the literature (78.6%, 33/42). We believe that focal TTF1 staining missed on TMAs is at most a minor contributor and does not fully explain the discrepancy. In a prior study comparing ER IHC expression in TMA cores versus whole slide sections, the disagreement for TMAs with two or three cores (approximately half of our cases were represented by two and half by three cores) versus whole section with a cutoff of ≥1% was 3.4% and 1.1%, respectively [31]. Therefore, the underestimation of TTF1 using TMAs should be less than 5%. Furthermore, TTF1 was highly prognostic in our analyses, despite using TMAs. Nevertheless, due to their high specificity GATA3 and TTF1 remain useful diagnostic markers in the differential diagnosis of MA and EC, whereas CD10 and calretinin are not.

We recommend a low threshold for ordering ancillary IHC on cases displaying features commonly observed in MA including blue low‐power appearance, luminal eosinophilic material, and admixture of architectural patterns such as tubules or sieve‐like and cuboidal cell shape. We also emphasize that the common solid/spindled patterns, which may not directly trigger consideration of an MA, should also prompt ancillary IHC. Features of exclusion are unequivocal squamous or prominent mucinous/seromucinous differentiation, except in an event of a distinct admixed Müllerian component. High‐grade nuclear atypia characteristic of p53 abnormal tumors should be absent [32]. At this point, we consider abnormal p53 and MMRd as being incompatible with a diagnosis of MA. However, exceptions may exist; e.g. the possibility of an acquired *TP53* mutation during progression as seen in many other cancer entities [33]. Euscher *et al* accepted one case with a *TP53* mutation and abnormal p53 IHC as an ovarian MA, which showed small foci of marked cytologic atypia [26]. On the other hand, MA should show normal PAX2 expression, while other markers (GATA3, TTF1, and ER/PR) have imperfect sensitivity and require complex integration with morphology.

Although case series of ovarian MA have been reported previously [26, 30], herein, we found the prevalence of MA among cases initially classified as EC to be 5.7%. While our cohorts are population‐based, their selection bias toward non‐high‐grade serous carcinomas and, therefore, the prevalence of MA within ovarian carcinomas as a whole could not be assessed. However, assuming that 10–15% of all ovarian carcinomas are EC [34], the frequency of MA among all ovarian carcinomas would be estimated to be 0.6–0.9%. This is comparable to a rate of 0.7% reported in two similar studies in endometrial carcinomas [35, 36] but lower than another study [37] again highlighting the application of different thresholds of diagnostic and exclusion criteria.

Two previous studies indicated an unfavorable prognosis for ovarian MA compared with other histotypes [26, 38]. However, our study is the first to show statistically significant and independent prognostic implications of MA histotype compared with EC, which remained significant in a stratified analysis for stage I tumors, highlighting the importance of recognizing MA at low stage. Overall, the survival curve for MA showed a gradual protracted decline similar to low‐grade serous carcinomas; with a notable crossing of clear cell carcinomas and an intermediate survival between EC and high‐grade serous carcinoma. The survival of MA is similar to p53 abnormal EC. Of note, when the initial FIGO grade was applied to MA, grade was not prognostic, supporting the previous notion that MA should not be graded [38].

A limitation of our study is the lack of *KRAS* mutation status, however, *KRAS* mutations are neither entirely sensitive (89%) nor specific (67%) for the distinction of MA from EC [39, 40]. Several examples considered as MA in our study showed a methylation profile consistent with MA providing an alternative gold standard beyond light microscopy and IHC and *KRAS* mutation status. Another limitation was the lack of full slide review for associations with other Müllerian lesions beyond endometriosis. MA with associated borderline tumors and as a part of mixed carcinomas have been reported in 12% and 42% of cases, respectively [26]. Future studies of MA and associated lesions using omics techniques might shed further light into whether transdifferentiation occurs as a continuum or a categorical switch. Since our inclusion criteria were only cases with a diagnosis of the five major histotypes of ovarian carcinomas, rare cases of MA carcinosarcoma, which historically may have been diagnosed as carcinosarcoma, could not have been identified [7, 26]. It is also important to keep in mind that the sensitivity and specificity of our IHC markers are somewhat artificial since they have been used to define the gold standard. While we used presence/absence for ER/PR and GATA3/TTF1 and diffuse or >50% for PAX2, these cutoffs should be further evaluated on full sections in the clinical setting.

Nevertheless, in the context of distinguishing MA from EC, PAX2 has value as a screening marker for MA due to its high sensitivity, while normal p53 and MMR proficiency are specific for MA. Classic MA morphology together with an MA IHC‐profile is diagnostic of MA. The occurrence of subtle MA morphology and non‐MA IHC profiles in some tumors requires complex integration to arrive at a final diagnosis. Future studies using methylation profiling and copy number analyses should clarify further gray areas such as double positive cases and different components in cases demonstrating MA admixed with Müllerian lesions.

## Author contributions statement

MK and C‐HL conceived, designed and supervised the study. EYK, ZA and MK identified MA cases, reviewed morphological features and collected IHC scores. SL, TO, TT, LW and NJPW participated in interobserver reproducibility. LSC, GSN, CJRS, AvD and FKFK provided resources including samples, clinical data abstraction and analysis tools. MK drafted the manuscript. All authors revised the manuscript and approved the final version.

## Supporting information


**Figure S1.** Flow chart of identification of 30 MA cases
**Figure S2.** PAX2 expression in normal Müllerian tissue
**Figure S3.** Illustration of MA#29 with exceptionally high ER expression (distribution 100%, intensity 3)
**Figure S4.** Kaplan–Meier survival analyses comparing MA and EC of NSMP stratified by stage
**Figure S5.** Hierarchical decision tree using combined morphologic and immunohistochemistry‐based identification of MA without PAX2
**Figure S6.** Kaplan–Meier survival analyses within EC of NSMP
**Figure S7.** Kaplan–Meier survival analyses, the same as Figure 4C, except for the addition of EC cases with morphological features suggestive of MA
**Figure S8.** Kaplan–Meier survival analyses within MA by stage, grade, TTF1 expression and GATA3 expression

**Table S1.** Expression of GATA3, TTF1, ER, PR, and PAX2 across histotypes (initial diagnoses)
**Table S2.** Interobserver reproducibility assessment by case
**Table S3.** Paired kappa values from the interobserver reproducibility assessment
**Table S4.** Summary of changes during immunohistochemistry integration in the interobserver reproducibility assessment
**Table S5.** Comparison of morphological features between mesonephric‐type and endometrioid carcinoma cases
**Table S6.** Multivariable survival analysis of mesonephric‐type versus endometrioid carcinoma cases

## Data Availability

Individual patient data and related tumor information underlying this article cannot be shared publicly due to data privacy protection laws. However, grouped data will be shared on reasonable request to the corresponding author.
