# Supplementary material for: Mesonephric‐type adenocarcinomas of the ovary: prevalence, diagnostic reproducibility, outcome, and value of PAX2
Source: J Pathol Clin Res. 2024 Jul 6;10(4):e12389. doi: 10.1002/2056-4538.12389 (PMC11227277; doi:10.1002/2056-4538.12389)
Supplement: Supplementary file 1 — Figure S1. Flow chart of identification of 30 MA cases Figure S2. PAX2 expression in normal Müllerian tissue Figure S3. Illustration of MA#29 with exceptionally high ER expression (distribution 100%, intensity 3) Figure S4. Kaplan–Meier survival analyses comparing MA and EC of NSMP stratified by stage Figure S5. Hierarchical decision tree using combined morphologic and immunohistochemistry‐based identification of MA without PAX2 Figure S6. Kaplan–Meier survival analyses within EC of NSMP Figure S7. Kaplan–Meier survival analyses, the same as Figure 4C, except for the addition of EC cases with morphological features suggestive of MA Figure S8. Kaplan–Meier survival analyses within MA by stage, grade, TTF1 expression and GATA3 expression [file CJP2-10-e12389-s001.pdf]

Mesonephric-type adenocarcinomas of the ovary: prevalence, diagnostic reproducibility, outcome, and value of PAX2

M Köbel *et al.*, *J Pathol Clin Res*, https://doi.org/10.1002/2056-4538.12389

Supplementary Figures S1–S8

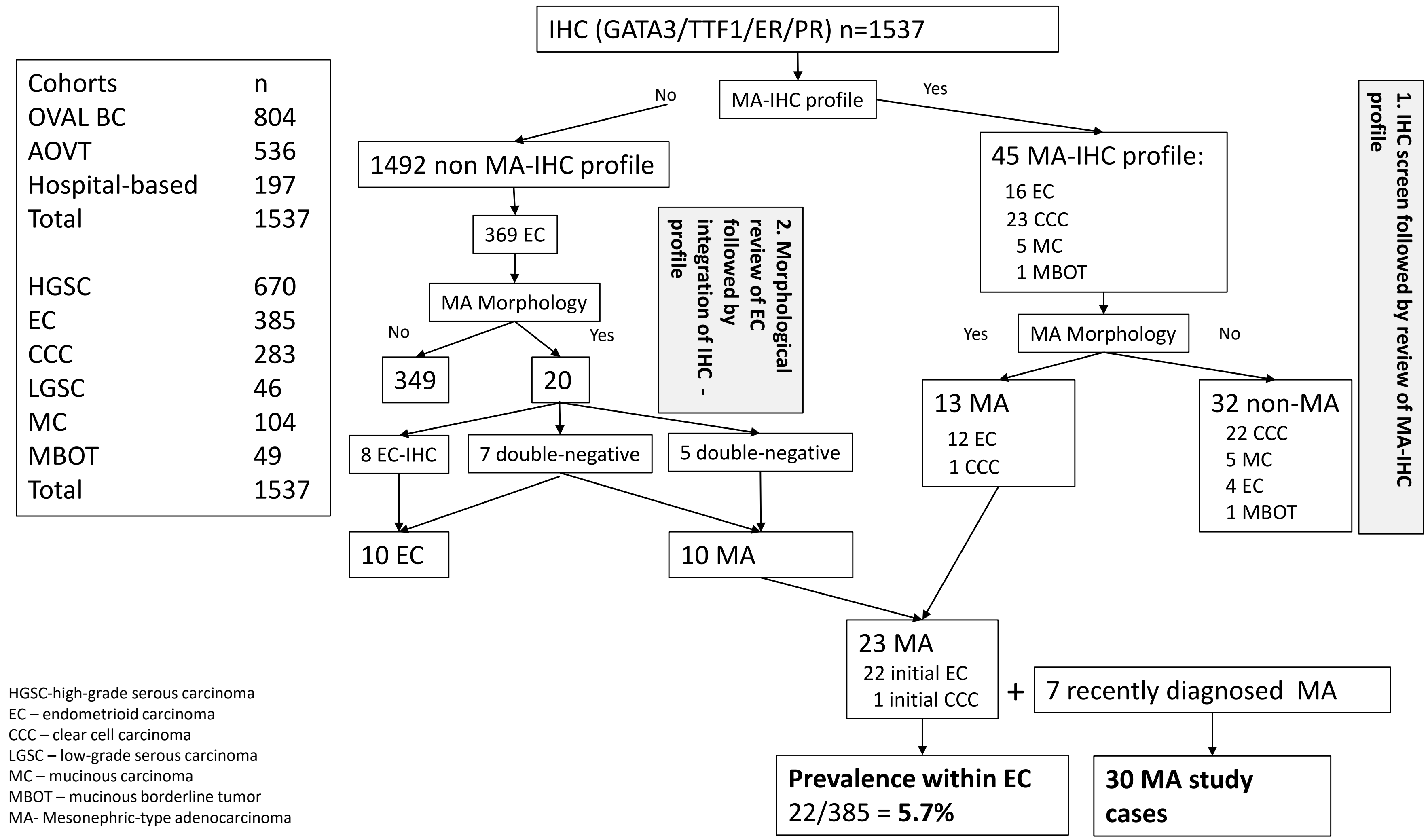

Figure S1. Flow chart of identification of 30 MA cases

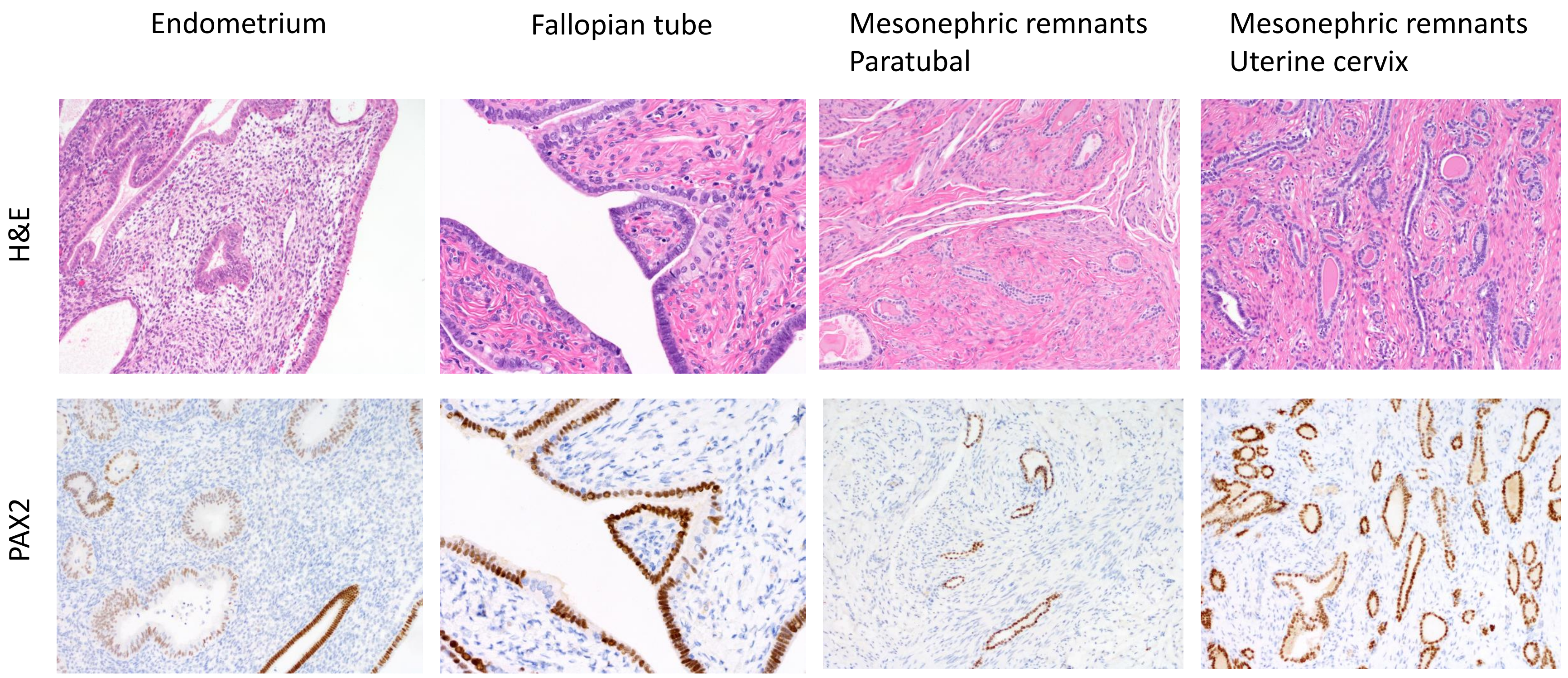

Figure S2. PAX2 expression in normal Müllerian tissue

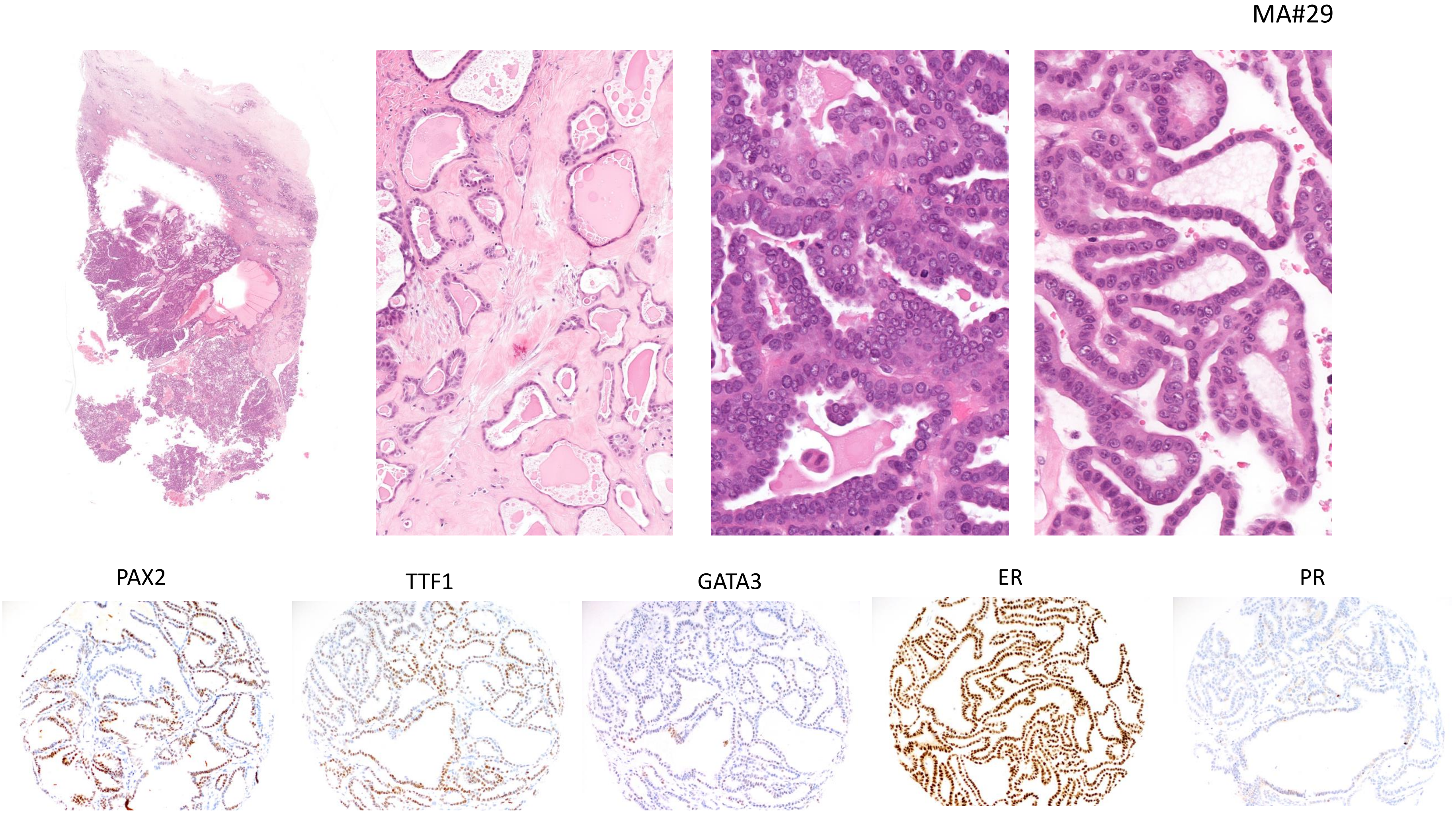

Figure S3. Illustration of MA#29 with exceptionally high ER expression (distribution 100%, intensity 3). Note, very focal PR expression (distribution 1%, intensity 1). PAX2 and TTF1 are expressed in >50% of tumor cells while GATA3 is focally expressed. The morphology is in keeping with an MA and the methylation profile of this case is consistent with MA.

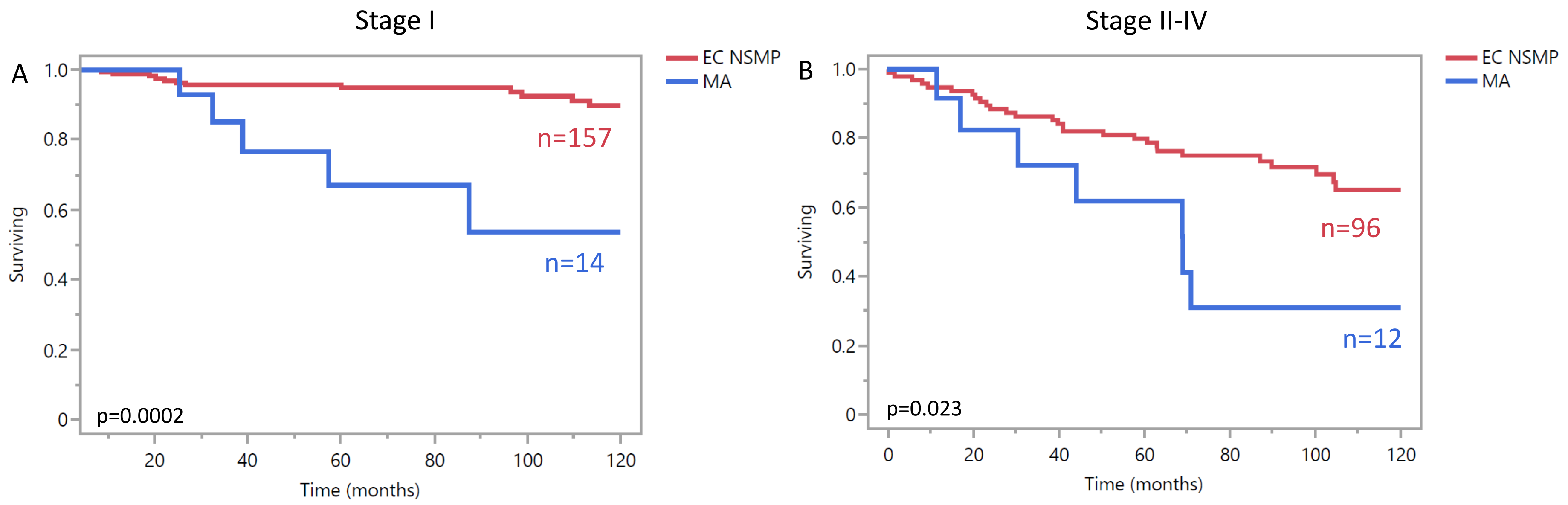

Figure S4. Kaplan-Meier survival analyses comparing mesonephric-type adenocarcinoma (MA) and endometrioid carcinoma (EC) of no specific molecular profile (NSMP) stratified by stage.

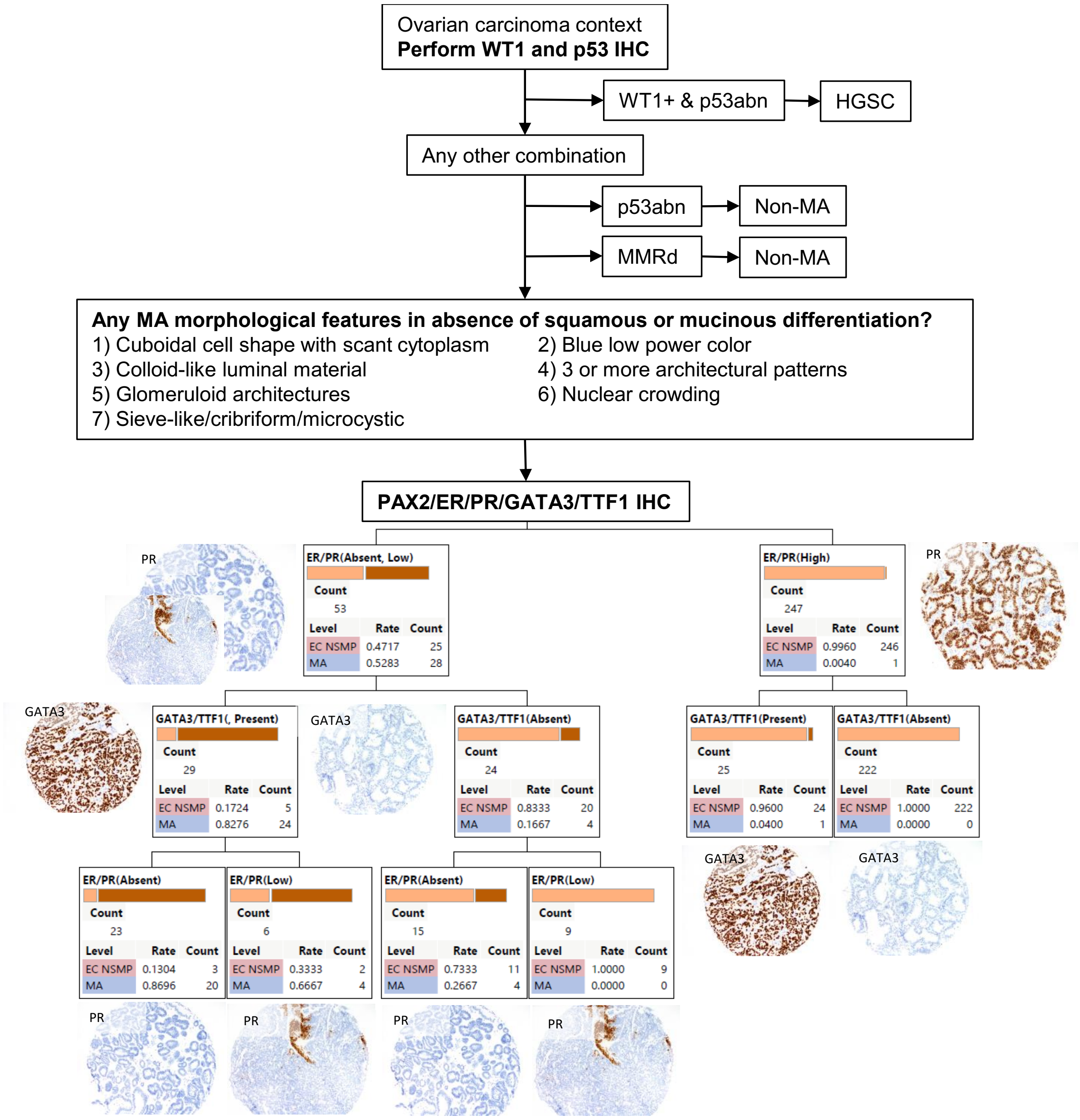

Figure S5 Hierarchical decision tree using combined morphologic and immunohistochemistry (IHC)-based identification of mesonephric-type adenocarcinoma (MA) without PAX2. First, a combination of WT1+ and p53 abnormal is highly specific for high-grade serous carcinoma (HGSC). Second, there are typical morphological features of MA; however, they can overlap with endometrioid carcinomas (EC). Third, to distinguish EC from MA, p53abn and MMRd molecular subtypes of EC are generally inconsistent with MA. Fourth, a traditional IHC panel consisting of ER/PR and GATA3/TTF1 to distinguish MA from EC, NSMP molecular subtype (if PAX2 is available please see Figure 5).

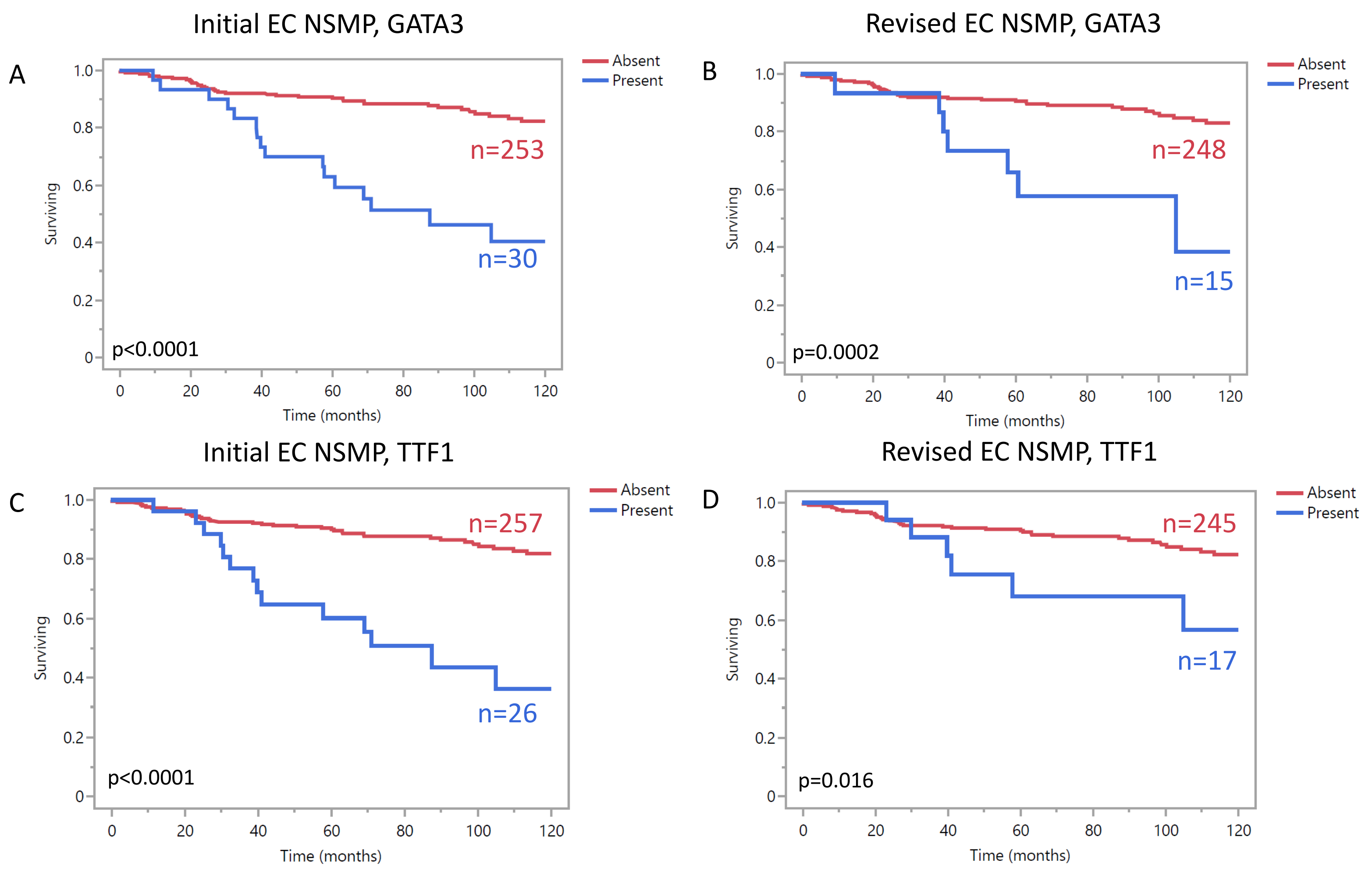

Figure S6. Kaplan-Meier survival analyses within EC of no specific molecular profile (NSMP): (A, B) by GATA3 expression from initial diagnosis and revised EC diagnosis after MA reclassification; (C, D) by TTF1 expression from the initial diagnosis and revised EC diagnosis after MA reclassification; (E, F) by combined ER/PR expression from initial diagnosis and revised EC diagnosis after MA reclassification; and (G, H) by PAX2 expression from initial diagnosis and revised EC diagnosis after MA reclassification.

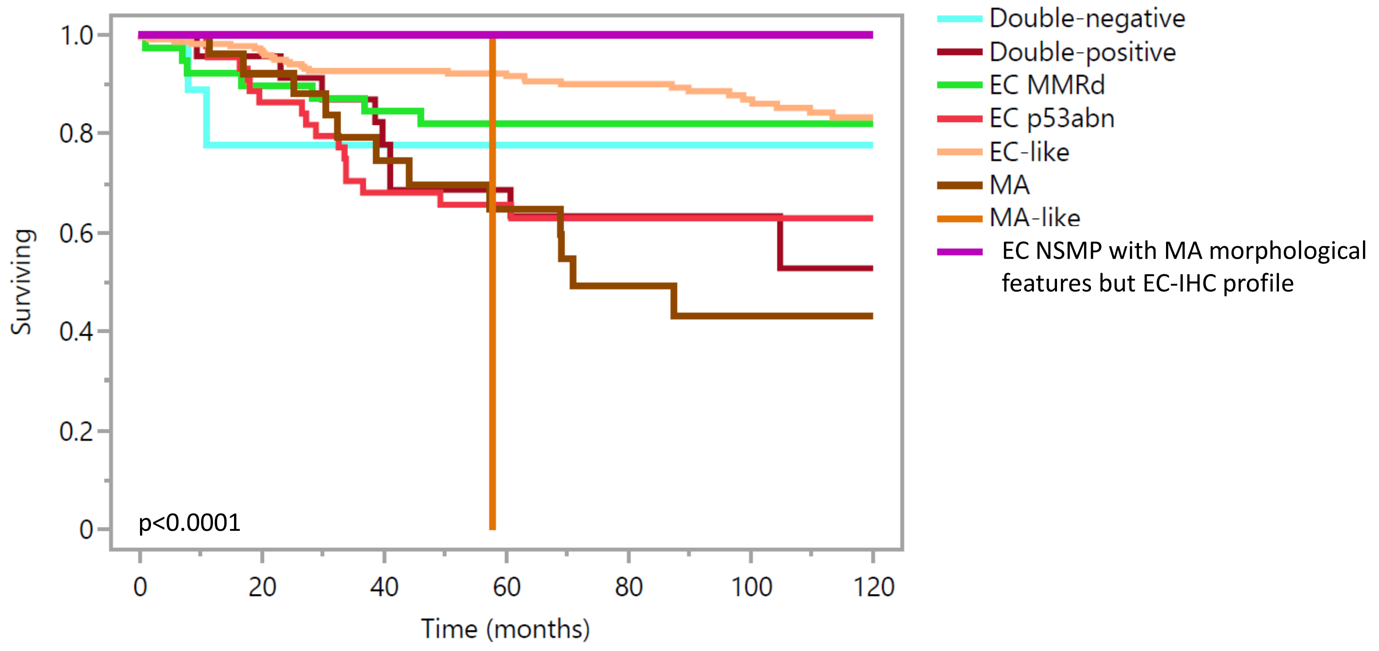

Figure S7. Kaplan-Meier survival analyses, the same as Figure 4C, except for the addition of EC cases with morphological features suggestive of MA.

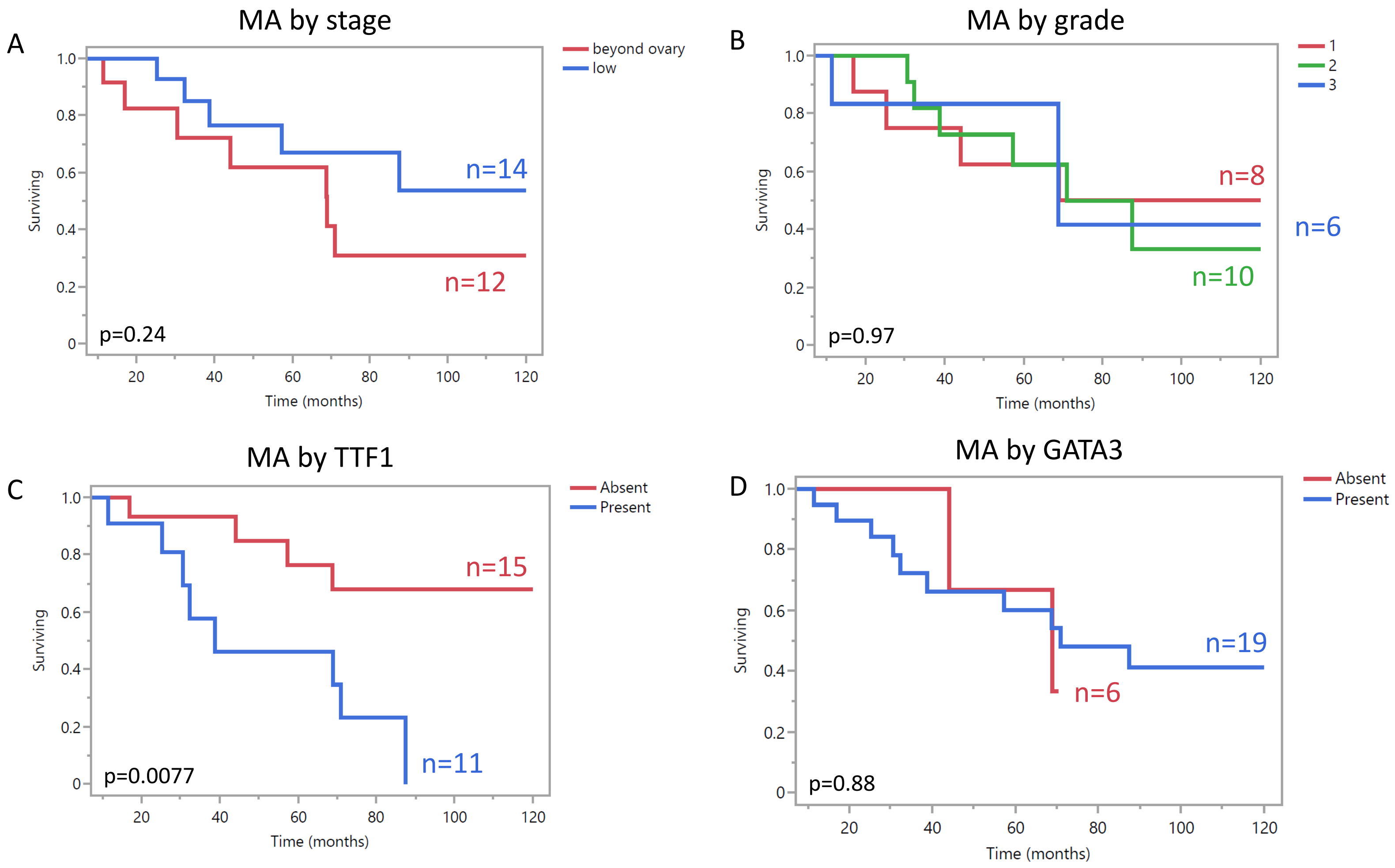

Figure S8. Kaplan-Meier survival analyses within MA: (A) by stage; (B) by grade; (C) by TTF1 expression; (D) by GATA3 expression.
